# The Target Differences of Anti-Tumorigenesis Potential of Curcumin and its Analogues Against HER-2 Positive and Triple-Negative Breast Cancer Cells

**DOI:** 10.34172/apb.2021.020

**Published:** 2020-11-07

**Authors:** Edy Meiyanto, Ulfatul Husnaa, Ria Fajarwati Kastian, Herwandhani Putri, Yonika Arum Larasati, Annisa Khumaira, Dyaningtyas Dewi Putri Pamungkas, Riris Istighfari Jenie, Masashi Kawaichi, Beni Lestari, Takashi Yokoyama, Jun-ya Kato

**Affiliations:** ^1^Cancer Chemoprevention Research Center, Faculty of Pharmacy, Universitas Gadjah Mada, Yogyakarta 55281 Indonesia.; ^2^Department of Pharmaceutical Chemistry, Faculty of Pharmacy, Universitas Gadjah Mada, Yogyakarta 55281 Indonesia.; ^3^Department of Pharmacology and Clinical Pharmacy, Faculty of Pharmacy, Universitas Gadjah Mada, Yogyakarta 55281 Indonesia.; ^4^Graduate School of Biological Sciences, Nara Institute of Science and Technology, 8916-5 Takayama-cho, Ikoma, Nara 630- 0192, Japan.; ^5^Division of Educational Development, Nara Institute of Science and Technology, 8916-5 Takayama-cho, Ikoma, Nara 630- 0192, Japan.; ^6^Laboratory of Tumor Cell Biology, Graduate School of Biological Sciences, Nara Institute of Science and Technology, Ikoma, Nara, 630-0101, Japan.

**Keywords:** Breast cancer, Curcumin analogues, HER2, Metastasis, NFκB, Triple-negative breast cancer

## Abstract

***Purpose:*** The current study aims to evaluate the* in vitro* cytotoxic and cell migration effects of synthetic curcumin and its analogues on HER2 and nuclear factor kappa B (NFκB) pathways, as well as the in vivo inhibitory effect on cancer growth of metastatic breast cancer.

***Methods:*** Cell viability, protein expression, and protein localization were determined *in vitro* using MTT assay, western blotting, and immunofluorescence, respectively. Meanwhile, scratch wound healing assay and gelatin zymography were conducted to investigate the metastasis inhibitory effect. The *in vivo* anti-tumor ability was evaluated in xenograft mouse model using triple-negative breast cancer (TNBC) cells.

***Results:*** Curcumin, PGV-0, and PGV-1 exhibited cytotoxic effect against HER2-overexpressing breast cancer cells. Although PGV-1 showed the best activity in the single cytotoxic assay, curcumin showed the strongest synergism with doxorubicin. Curcumin and PGV-0 inhibited membrane localization of HER2. In contrast, PGV-1 neither inhibited localization nor decreased the expression of HER2, nonetheless showed the most potent inhibition against nuclear localization of p65 indicating the different mechanisms of curcumin, PGV-0, and PGV-1. Regarding cancer metastasis, curcumin and PGV-1 showed inhibitory activities against cell migration and inhibited MMP-2 and MMP-9 protein expression. Lastly, PGV-1 was more potent compared to curcumin to suppress the tumor formation of metastatic breast cancer xenograft model in nude mice.

***Conclusion:*** Overall, our study strengthens the potency of curcumin analogue, PGV-1, for treating several types of cancer, including metastatic breast cancer.

## Introduction

**Figure 1:**
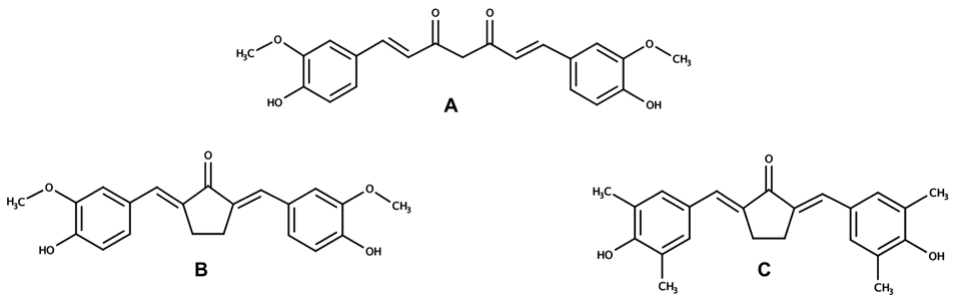
The structures of curcumin and its analogues. A. Curcumin , B. PGV-0 , C. PGV-1

Curcumin ((1E,6E)-1-(4-hydroxy-3-methoxyphenyl)-7-(3-methoxy-4-methylphenyl) hepta-1,6-diene-3,5-dione) (Figure 1A), a symmetric molecule isolated from turmeric (*Curcuma longa*), exerts potent chemopreventive activities via numerous mechanisms including interfering cell cycle signaling pathway, inhibiting cancer metastasis, and modulating redox homeostasis in cancer cells.^1-4^ Despite its superior potency, curcumin has not been available for clinical cancer therapy due to its chemical instability and poor bioavailability.^5^ Namely Pentagamavunon-0 (PGV-0) ((2E,5E)-2-[(4-hydroxy-3-methoxyphenyl) methylidene]-5-[(3-methoxy-4-methylphenyl) methylidene] cyclopentane-1-one) (Figure 1B) and Pentagamavunon-1 (PGV-1) ((2E,5E)-2-[(4-hydroxy-3,5-dimethylphenyl) methylidene] -5-[(3-methoxy-4,5-dimethylphenyl) methylidene]cyclopentan-1-one) (Figure 1C), the curcumin analogues were synthesized by the Faculty of Pharmacy Universitas Gadjah Mada, differ from curcumin in their chemical core in order to improve the physicochemical properties of curcumin.^6^ Both PGV-0 and PGV-1 have been demonstrated to exhibit better cytotoxic and anti-inflammatory activities compared to curcumin.^7,8^ Our previous study showed that PGV-0 and PGV-1 possess cytotoxic activities and augment the sensitivity of resistant breast cancer cells to doxorubicin.^9^ In this concern, we also analyzed the hypothetical interaction between PGV-0 or PGV-1 and HER2 receptor under molecular docking, showing that those compounds bind to HER-2 receptor at ATP binding site. In addition, PGV-0 and PGV-1 also strengthen the cytotoxicity of 5-fluorouracil in colon cancer cells.^10,11^ In general, PGV-1 performed most potent to inhibit cancer cell growth. Recently, PGV-1 showed a strong inhibitory effect against 4T1 cells, a triple-negative breast cancer (TNBC) cell model, under *in vitro* experiment through ROS generation.^12^ These studies warrant the potency of PGV-0 and PGV-1 to be further investigated as anticancer drugs, especially to the specific types of breast cancers, namely HER2 positive and TNBC.

HER2 positivity (HER2^+^), a subtype of breast cancer characterized by either HER2 protein overexpression or gene amplification, is found in approximately 15%–30% of breast cancers and contributes with low survival and poor clinical outcome in breast cancer patient.^13^ This reduced survival is closely related to aggressive proliferation of HER2-overexpressing cells^14^; as well as the occurrence of breast cancer metastasis as HER2 overexpression increases the invasive potential of cancer cells. HER2 activates multiple signaling axes, especially in MAPK signaling system and STAT3; leading to rapid tumor proliferation and cancer metastasis.^15^ HER2 also promotes epithelial-mesenchymal transition (EMT) by upregulating TGFβ/SNAIL-ZEB1, activates a Wnt-dependent EMT-like dissemination program, and downregulates E-cadherin expression.^16,17^ Interestingly, the increase of HER2 expression could be observed in metastatic cancer cells even though the primary tumor is HER2 negative.^15,18^

In addition, HER2 signaling shows crosstalk with nuclear factor kappa B (NFκB) pathway. HER2 activates NFκB pathway primarily through IκB kinase-α (IKKα), differing from the ordinary activation of NFκB that is primarily through IKKβ.^19^ Reciprocally, NFκB regulates the expression of HER2 through its binding to the promoter of the *HER2* gene.^20,21^ As a powerful transcription factor, NFκB also activates the expression of myriads genes involving in proliferation, invasion, metastasis, and EMT.^22^ Further, constitutive activation of NFκB in HER2^+^ cells causes resistance to anti-HER2 drugs.^23^ Accordingly, HER2 and NFκB signaling are promising pathways to target the aggressive proliferation and metastasis of breast cancer.

In this study, we utilized HER2-overexpressing breast cancer cells, MCF7/HER2-5, as a model to evaluate the activities of synthetic curcumin analogues, PGV-0 and PGV-1, in HER2 and NFκB signaling pathways. In addition, we also employed 4T1 cells for *in vivo* tumor implanted model representing the TNBC tumor model. The result of this study is expected to provide more comprehensive evidence of PGV-0 and PGV-1 to be developed as HER2^+^ or HER2-breast cancer therapy and for more comprehension of TNBC.

## Materials and Methods

### Chemicals and cell lines

Curcumin, PGV-0, and PGV-1 were synthesized by Curcumin Research Center (CRC), Faculty of Pharmacy, Universitas Gadjah Mada. All cell lines were the collection of Nara Institute of Science and Technology (NAIST), Japan. Mammary carcinoma cells, MCF-7/HER2-5 and MCF-7/mock cells were kindly given by Prof. Yoshio Inouye (Department of Surgery, Toho University School of Medicine). HEK293T cells and 4T1 cells were obtained from Prof. Masashi Kawaichi (NAIST). All cells were maintained in Dulbecco’s modified Eagle medium (DMEM) medium at 37 °C supplemented with 10% FBS (Sigma), HEPES (Sigma), sodium bicarbonate (Sigma), 150 IU/mL penicillin (Gibco), and 150 µg/mL streptomycin (Gibco), and 1.25 µg/mL fungizone (Gibco).

### Cytotoxic assay

MCF-7/HER2-5, MCF-7/mock, and HEK293T cells were plated in 96-well plates at a density of 1 × 10^4^ cells per well and allowed to grow for 24 h. The cells were then treated with a series concentration of curcumin, PGV-0, and PGV-1 for 24 h. After treatment, 5 mg/mL of 3-(4,5-dimethylthiazol-2-yl)-2,5-diphenyltetrazolium bromide (MTT, Sigma) in PBS was diluted in DMEM (1:9), 100 µL of the reagent was added to each well, and incubated for 3 h at 37 °C. The reaction of MTT formazan was stopped by 10% sodium dodecyl sulfate (SDS) in 0.01 N HCl followed by incubation overnight at room temperature. To absorbance at λ 595 nm was measured using an ELISA microplate reader.

### Immunofluorescence

MCF-7/HER2-5 cells were seeded in glass coverslips inside the 6-cm dish and cultured to 80% confluence. The cells were then treated with compounds, either alone or in combination with doxorubicin, and incubated for 24 h. After 24 h, the cells were fixed using 70% ethanol and incubated for 15 min at room temperature. After rinsing with PBS, the cells were incubated with the 1% BSA as blocking serum for 30 min at room temperature. Then, the cells were incubated with a primary antibody [mouse anti-p65 (Santa Cruz) or mouse anti-HER2 (Santa Cruz)] overnight at 4°C followed by secondary antibody (Santa Cruz) for 1 h. Then, DAPI staining was used to label nuclei for 10 min at room temperature in the dark. The cells were again rinsed with PBS and placed on sliding glass. The slides were analyzed and photographed using a fluorescence microscope.

### Western blotting

MCF-7/HER2-5 cells (2 × 10^5^ cells/mL) were grown to confluence and treated with PGV-1 or curcumin at the indicated concentration for 24 h. After treating with the compounds, the cells were washed with cold PBS. The cells were then lysed in lysis buffer containing 50 mM Tris-HCl (pH 8.0), 150 mM sodium chloride (NaCl), 0.1% SDS, 1.0% NP-40 mM, 100× protease inhibitor cocktail, and 100 mM phenylmethylsulfonyl fluoride in propanol and incubated for 10 min at 4°C. Cells were scraped using a scrapper on the ice and added to a microtube. The cell suspension was sonicated for 10 s and centrifuged at 15 000 rpm for 20 min at 4°C. The supernatant (cell lysate) was used as the protein sample for western blotting. The protein samples were denatured in 5× SDS-PAGE sample buffer and subjected to 10% SDS-polyacrylamide gel electrophoresis. The separated proteins were transferred onto a PVDF membrane, followed by blocking with 5% skim milk powder (w/v) in TBS (20 mM Tris and 133 mM NaCl, 1 N HCl) containing 20% Tween 20 for 1 h at room temperature. The membrane was probed for the protein expression levels using a specific primary antibody of mouse anti-HER2 (Santa Cruz) overnight at 4°C. Equal protein loading was examined by tubulin detection using a mouse anti-tubulin-specific antibody (Santa Cruz). After washing with 1× TBS-T, the membrane was probed with an HRP-conjugated appropriate secondary antibody for 1 h at room temperature and visualized by ECL PRIME or the ECL detection system.

### Gelatin zymography

MCF-7/HER2-5cells (2 ×10^5^) on 6 well plate was incubated for 24 h in CO_2_ incubator then treated with curcumin or PGV-1 for 24 h. The media were collected and subjected to gel electrophoresis (SDS-PAGE) using 10 % gel containing 0.1% gelatin running at 100 volts, 50 A for 2 h. The gel was renatured with renaturing solution containing 2.5% Triton X-100 for 30 min and incubated with incubation buffer for 20 h. The gel was then stained with 0.5% Coomassie Brilliant Blue and incubated for 30 min. After 30 min, the gel was de-stained by a destaining solution. The gel is scanned and documented. Band intensities were measured using ImageJ software.

### Scratch wound healing assay

MCF-7/HER2-5 cells (8 ×10^4^) were seeded into each well of 24 well plates and incubated for 24 h. Cells were then starved by changing the medium into serum-free medium for 18 h. Cells then were scratched with a yellow tip and treated with curcumin, PGV-1, doxorubicin, or the combination treatment. Following the treatment, cells were documented at 0, 24, 48 h. Quantification of the area of wound closures were calculated using ImageJ software.

### In vivo experiment

Nude mice bearing metastatic breast cancer cells were generated by subcutaneously (s.c.) implantation of 4T1 cells (5 × 10^4^ cells in 0.2 mL medium) into the four flanks of 6-week-old nude mice on day 0. Starting from the same day, treatments were given with curcumin and PGV-1 (25 mg/kg BW) in corn oil via intra-peritoneal (i.p) administration every 2 days for 12 days. The untreated mice received corn oil as a vehicle. Tumor sizes were measured during this period.

### Data Analysis

The data analysis were conducted according to the previously reported.^9,23^ All statistical analyses were performed using ANOVA in SPSS 16 software with *P* value of 0.05 as considered to be significantly different.

## Results

### Cytotoxic activity of curcumin analogues against MCF-7/HER2-5, MCF-7/mock, and HEK293T cells

**Figure 2:**
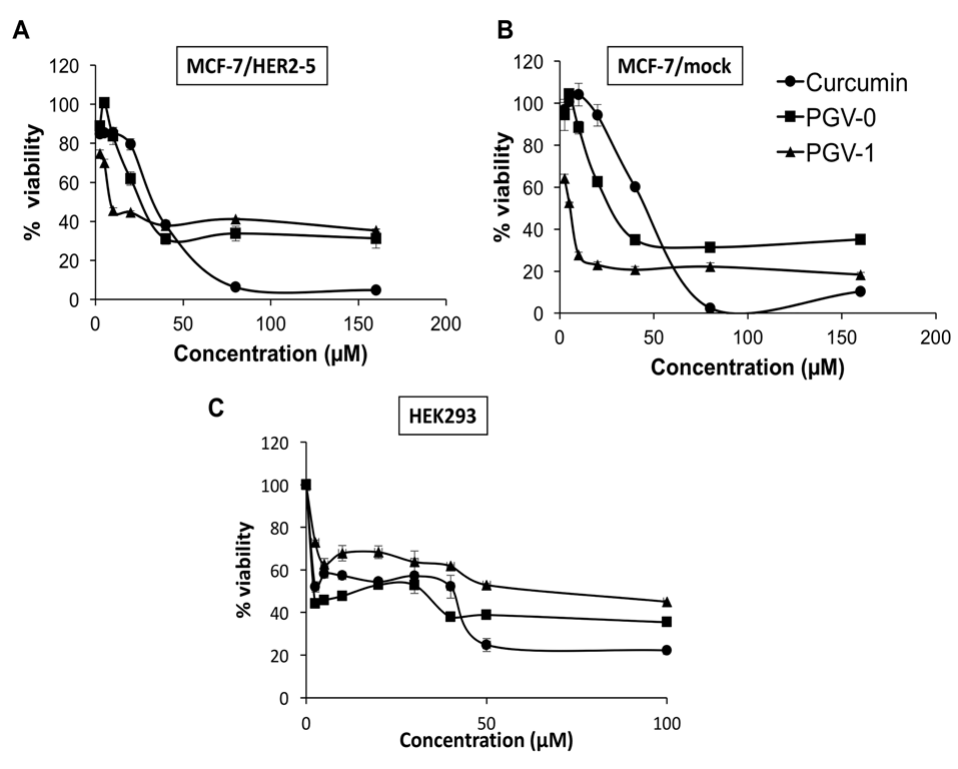
Cytotoxic effects of curcumin analogues on MCF-7/HER2-5 cells, MCF-7/mock cells, and HEK293T cells. MCF-7/HER2-5, MCF-7/mock, and HEK293 cells (1 × 10^4^ cells/well) were seeded in a 96-well plate, incubated for 24 h, and then treated with curcumin, PGV-0, or PGV-1. Cell viability was determined using MTT assay as described in the methods. A graph was plotted between concentration and cell viability of curcumin analogues on A. MCF-7/HER2-5 cells, B. MCF-7/mock cells, and C. HEK293T cells. The cytotoxicity of curcumin analogues was expressed by percent cell viability and is shown as the mean ± SE of three experiments. The IC50 values were obtained from the calculation of the linear regression of concentration vs % cell viability with *P* < 0.05.

A single treatment of curcumin, PGV-0, and PGV-1 on MCF-7/HER2-5 and MCF-7/mock showed cytotoxic effects (Figure 2A and 2B) with IC_50_ values as shown in Table 1. The IC_50_ of curcumin, PGV-0, and PGV-1 were 52, 32, 14 μM on MCF-7/HER2-5 and 38, 18, 5 μM on MCF-7/mock, respectively. The compounds resulted in higher IC_50_ values on MCF-7/HER2-5 cells, indicating that the transfection of HER2 genes altered the cell response to the compounds. Among the other compounds, PGV-1 exhibited the strongest cytotoxicity on both mammary carcinoma cells.

**Table 1:** IC_50_ value of curcumin analogues on MCF-7/HER2-5 and MCF-7/mock cells

**Compound**	**IC**_50_		
	**MCF-7/HER2-5**	**MCF-7/mock**	**HEK293T**
Curcumin	52 µM	38 µM	40 µM
PGV-0	32 µM	18 µM	30 µM
PGV-1	14 µM	5 µM	78 µM

Selectivity is an important property of desired chemotherapeutic agents, in which a compound should exhibit higher potency against cancer cells compared to normal cells. Mathematically, selectivity is a ratio that measures the window of cytotoxicity in normal cells, with selectivity index (SI) >3 represents good selectivity of a compound.^24^ Hence, we also checked the cytotoxic activity of compounds on HEK293T cells (kidney cells that represented normal cell model) and calculate their SI. As shown in Figure 2C and Table 2, only PGV-1 exhibited SI > 3 in both MCF-7/HER2-5 and MCF-7/mock cells, suggesting its good selectivity and potency to be considered as a chemotherapeutic agent.

**Table 2:** Selectivity index (SI) of curcumin analogues

**Cell type**	**SI**		
	**Curcumin**	**PGV-0**	**PGV-1**
MCF7/mock	1.05	1.67	15.60
MCF7/HER2-5	0.77	0.94	5.57

### Cytotoxic effect of the combination of curcumin analogues with doxorubicin on MCF-7/HER2-5 cells

**Figure 3:**
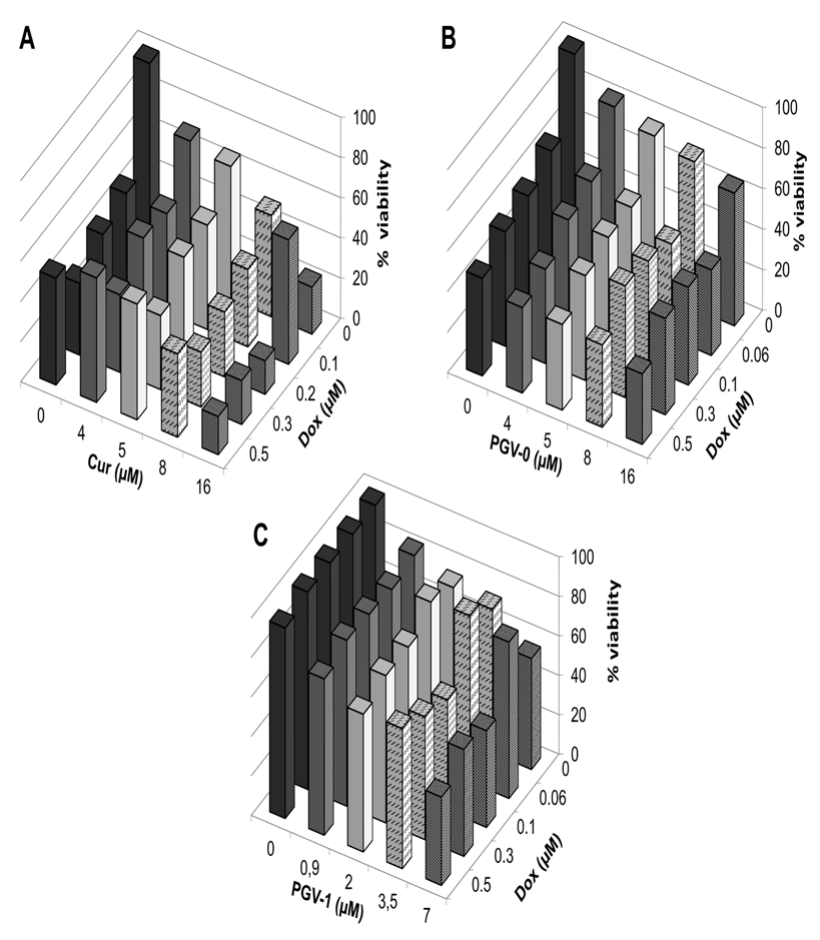
Combinational cytotoxic activity of curcumin and its analogues against MCF-7/HER2-5 cells. MCF-7/HER2-5 cells (1 × 10^4^ cells/well) were seeded in a 96-well plate, incubated for 24 h, and then treated with a combination of doxorubicin with A. Curcumin, B. PGV-0, and C. PGV-1. Cell viability was determined using MTT assay as described in the methods. The graph is plotted using the mean values from three replications.

Here, we used doxorubicin that known to be the standard chemotherapy for breast cancer as a threat against several adverse effects and raising resistance. Therefore, the application of combinatorial therapy is desirable to increase the effectivity of doxorubicin. A combinatorial cytotoxic assay was conducted by combining the curcumin analogues and doxorubicin on MCF-7/HER2-5 cells with serial concentrations of 1/20, 1/8, 1/4, and 1/2 of the IC_50_ values of both compounds. The combination index (CI) value was calculated to evaluate the synergistic effect of the combination. A synergistic effect is indicated by a CI value <0.9. The result showed that the three compounds enhanced the cytotoxicity of doxorubicin (Figure 3). In addition, the combination of curcumin and doxorubicin showed the lowest cell viability among the other combinations, suggesting that curcumin and doxorubicin was the best combination. Furthermore, the CI value confirmed that the combination of curcumin and doxorubicin at all doses was synergistic and resulted in the lowest CI value among all combinations (Tables 3,4, and 5). This finding suggests that curcumin was the best combination with doxorubicin.

**Table 3:** CI value of combinational cytotoxic assay of curcumin and doxorubicin

**Curcumin(µM)**	**Doxorubicin (µM)**			
	0.1	0.2	0.3	0.5
4	0.11	0.16	0.16	0.35
5	0.14	0.18	0.17	0.34
8	0.16	0.17	0.19	0.30
16	0.45	0.23	0.27	0.31

**Table 4:** CI value of combinational cytotoxic assay of PGV-0 and doxorubicin

**PGV-0 (µM)**	**Doxorubicin (µM)**			
	0.06	0.1	0.3	0.5
4	0.23	0.21	0.21	0.25
5	0.23	0.25	0.28	0.27
8	0.23	0.32	0.44	0.31
16	0.35	0.48	0.54	0.38

**Table 5:** CI value of combinational cytotoxic assay of PGV-1 and doxorubicin

**PGV-1 (µM)**	**Doxorubicin (µM)**			
	0.06	0.1	0.3	0.5
0.9	0.60	0.74	1.04	0.94
2	1.47	0.87	1.03	0.88
3.5	2.80	0.43	0.74	1.41
7	3.94	0.51	0.80	0.52

### Effect of curcumin analogues on the expression and localization of HER2

**Figure 4:**
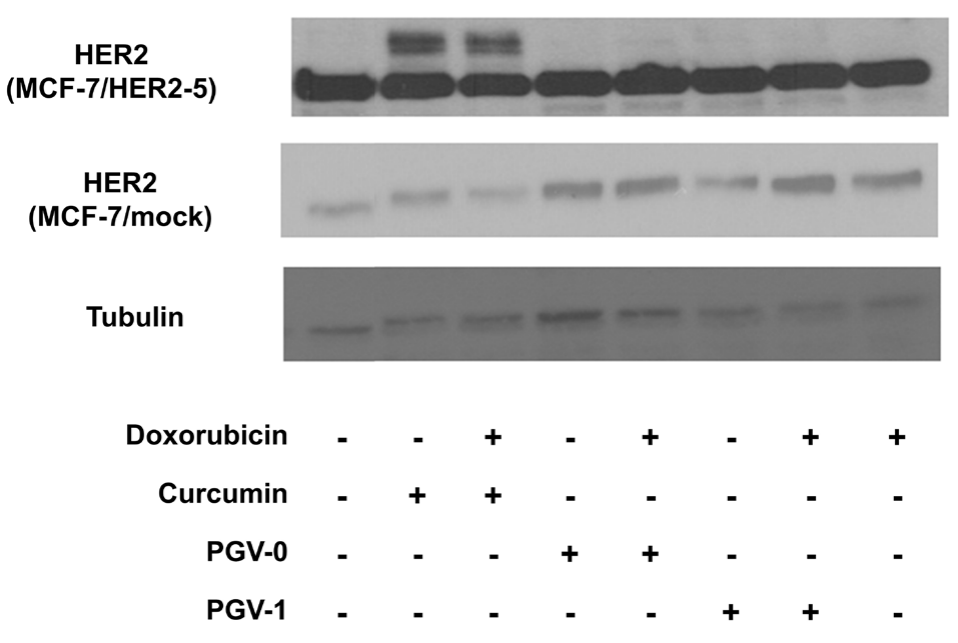
The effect of curcumin and its analogues on HER2 expression in MCF-7/HER2-5 cells analyzed by western blotting. Cells were treated with ½ IC50 value of each of the single compound and its combination with doxorubicin for 24 h and then analyzed using western blotting as described in the methods.

To confirm whether the cytotoxic activity of curcumin analogues was related to HER2 overexpression of MCF-7/HER2-5 cells, the expression of HER2 was observed by western blotting (Figure 4). MCF-7/HER2-5 cells exhibited a higher expression of HER2 compared to the mock cells. Although the cytotoxic effect of PGV-1 was more potent than those of curcumin and PGV-0, PGV-1 did not decrease the expression of HER2. Furthermore, curcumin decreased the levels of 185-kDa HER2 and exhibited two additional bands, which are interesting for further investigation.

**Figure 5:**
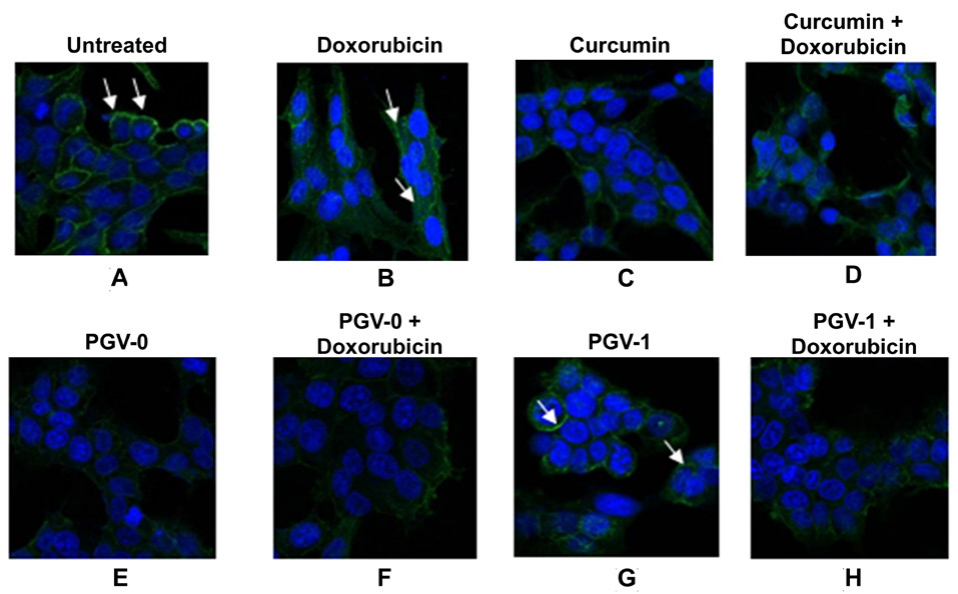
The effect of curcumin and its analogues on HER2 expression in MCF-7/HER2 cells. Cells were treated with ½ IC50 value of each of the single compound and its combination with doxorubicin for 16 h and then analyzed by immunofluorescence. A. Untreated cells with antibody HER2; cells treated with B. Doxorubicin, C. Curcumin, D. Curcumin+Doxorubicin, E. PGV-0, F. PGV-0+doxorubicin, G. PGV-1, and H. PGV-1+doxorubicin. Arrows indicate the localization of HER2

Immunofluorescence staining revealed that curcumin, as well as PGV-0 inhibited HER2 localization to the plasma membrane (Figure 5). On the other hand, PGV-1 and its combination with doxorubicin had no effect on the localization of HER2 protein on MCF-7/HER2-5 cells. These findings revealed the different mechanisms of PGV-1 effects than those of curcumin and PGV-0.

### Effect of curcumin analogues on p65 localization

**Figure 6:**
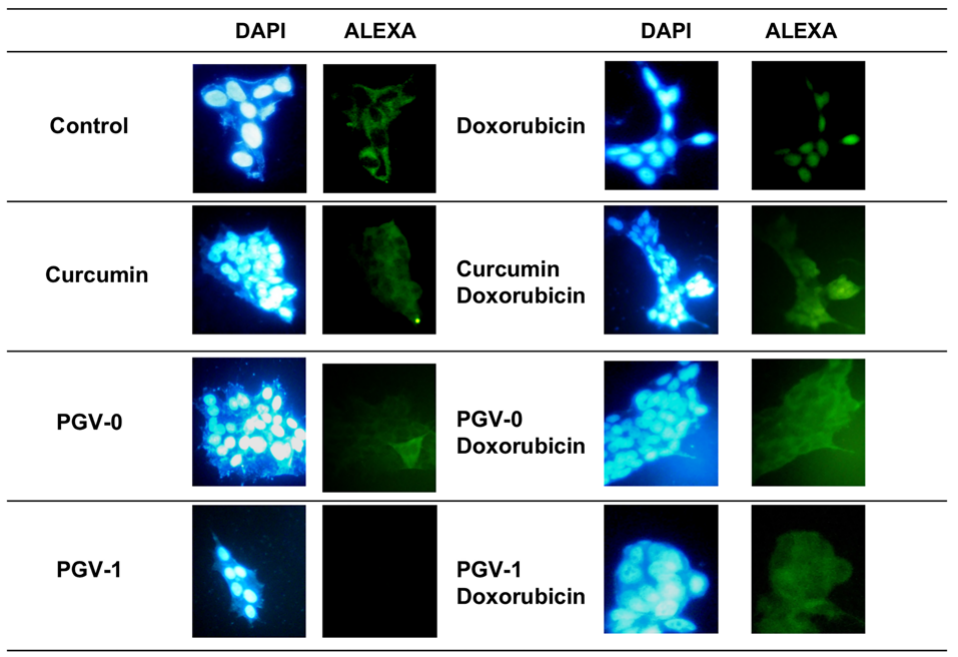
Effect of curcumin and its analogues on p65 localization in MFC-7/HER2-5 cells. Cells were grown on coverslips and treated with compounds as indicated with the concentration of ½ IC50 and then subjected to immunofluorescent staining with p65 antibody and DAPI as described in the materials and methods. Visualizations were performed under a fluorescence microscope with 400× magnification.

The NFκB pathway has been reported to be downstream of HER2 and regulates numerous cellular functions, including those involved in tumorigenesis.^19^ Therefore, we examined the effect of curcumin analogues on the activation of p65, an active subunit of NFκB, as shown in Figure 6. We found the more localized p65 in the nucleus in doxorubicin treatment, indicating the activation of p65 occurred. Curcumin and its analogues decreased the nucleus expression of p65 and PGV-1 showed the lowest nucleus localization of this protein. This was an interesting finding, considering that PGV-1 did not decrease the expression or the localization of HER2. Thus, the effect of PGV-1 on the localization of p65 was not related to HER2 expression, but maybe due to the inhibition of HER2 activation as reported previously.^9^ In this regard, PGV-1 showed a unique effect compared to curcumin and PGV-0 and needs for further investigation.

### Effect of curcumin and PGV-1 on cancer metastasis

**Figure 7:**
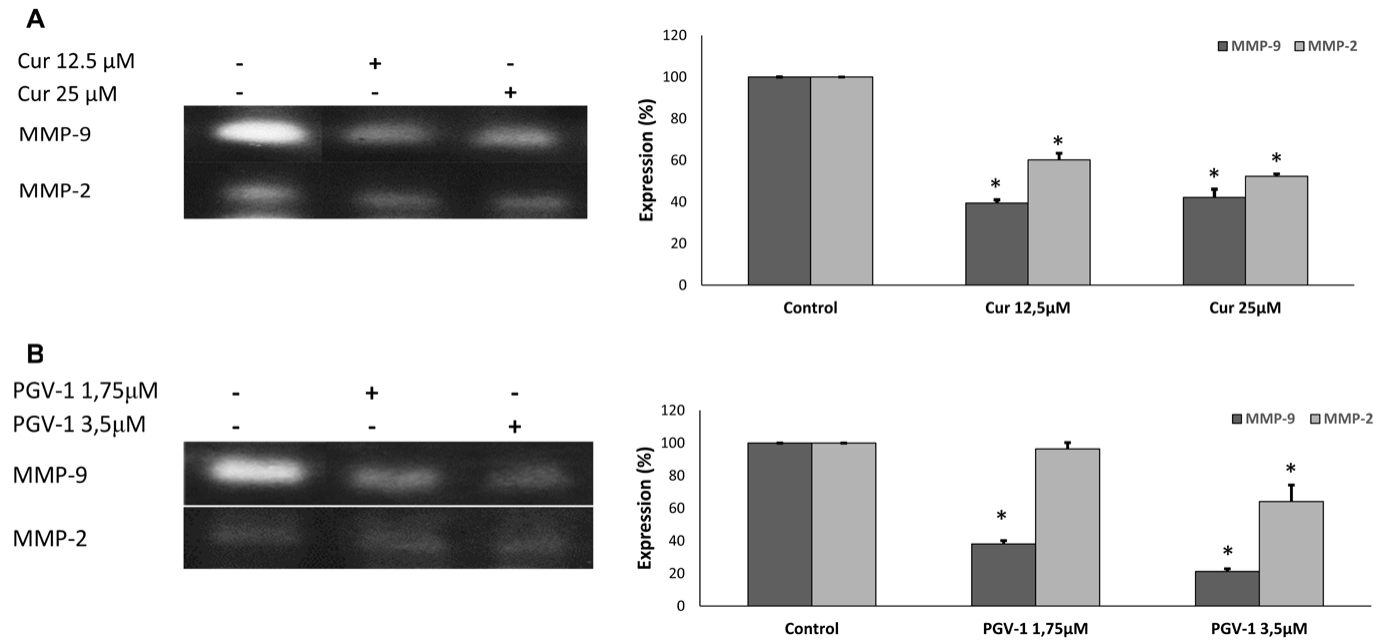
The effect of curcumin and PGV-1 on the secretion of MMP-2 and MMP-9. The MCF-7/HER2 cells (2 x 10^5^ cells/mL) treated with PGV-1 or curcumin at the indicated concentration for 24h. The band profiles of MMP-2 and MMP-9 expression in (A) curcumin treatment and (B) PGV-1 treatment. The band intensities were quantified using ImageJ (n=3).

Cancer metastasis involves multistep processes, including cancer cell invasion to surrounding tissue and cell migration.^25^ Matrix metalloproteinase proteins (MMPs), especially MMP-2 and MMP-9, are key proteins in invasion as these proteins degrade components of extracellular matrix, allowing the cancer cells to invade their surrounding tissues.^26^ Hence, we checked the effect of PGV-1 and curcumin on the expression of MMP-2 and MMP-9 using gelatin zymography (Figure 7). We found that both curcumin and PGV-1 decreased the level of MMP-2 and MMP-9.

**Figure 8:**
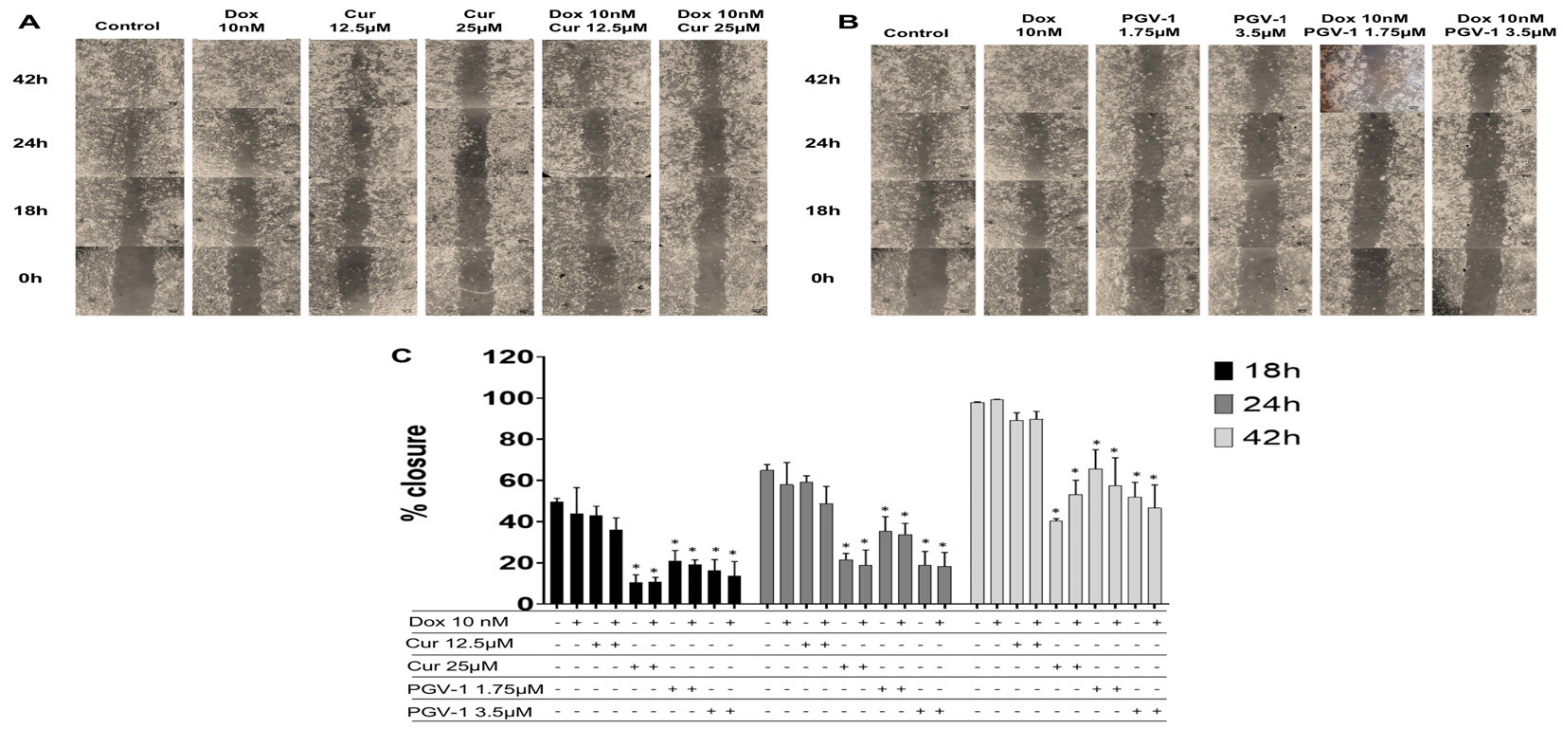
The effect of curcumin and PGV-1 on cell migration. The MCF-7/HER2 cells (7.5 × 10^5^ cells/mL) were scratched then treated with PGV-1 or curcumin at indicated concentration alone and in combination with doxorubicin (10 nM). A and B. The morphology of the cells after scratched and treated with the compounds for 0, 24, and 48 h under an inverted microscope with magnification of 100 x. C.
The percentage of 4T1 cells closure after treatment. The area of closure was measured using ImageJ (n=3) and % closure was calculated. Asterisk (*) indicate statistical difference compared to % closure of control (untreated cells) at the same hour of observation (*P*  < 0.05).

We then checked the effect of compounds on cell migration using scratch wound healing assay to evaluate the potential inhibitory effect of PGV-1 in cancer metastasis (Figure 8). We used doxorubicin at a low dose as an inducer of cell migration and we found that doxorubicin did not inhibit cell migration. Meanwhile, curcumin only inhibited cell migration at higher dose (25 μM). On the other hand, PGV-1 exhibited an inhibitory effect on cell migration at a dose as low as 1.75 μM. Combination of curcumin or PGV-1 with doxorubicin showed similar results with the treatment of each compound alone. These results suggest that curcumin or PGV-1, but not doxorubicin, was the key inhibitor of cell migration and PGV-1 still performed a superior effect.

### Effect of curcumin and PGV-1 on tumor progression in vivo

**Figure 9:**
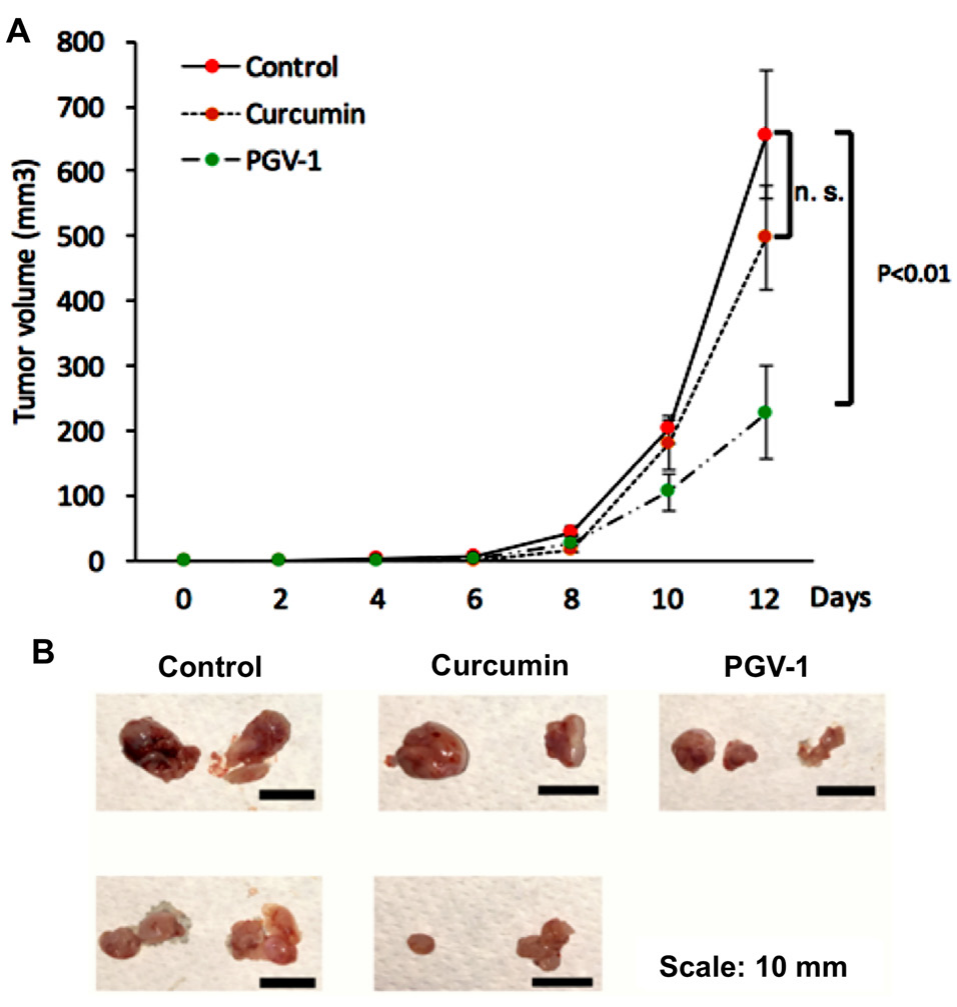
The effect of curcumin and PGV-1 on xenograft mouse model. 4T1 metastatic breast cancer cells (5 × 104 cells) were transplanted s.c. into the flanks of mice. Mice were then treated with curcumin or PGV-1 (25 mg/kg BW) in corn oil were administered to mice (50 mL of 10 mg/mL curcumin for 20 g BW) via i.p. injection every two days for 12 days and tumor sizes were recorded during this period. After 12 days, mice were sacrificed and tumors were taken. A. Tumor sizes (mm) were measured every 2 days. B. Representation of tumor taken at the end of experiment for each treatment.

Since we did not find significant differences of the PGV-1 treatment on HER expression profiles, we then evaluated the efficacy focusing on PGV-1 against tumor xenograft using 4T1 cells, a highly tumorigenic HER2- and metastatic cell line, which are suitable for our purpose in developing an anti-metastatic agent.^27^ We used curcumin as the comparison. Previously, we found that PGV-1 exhibited strong *in vitro* cytotoxic effect to 4T1 cells with the IC_50_ value of 4 µM and inhibited cell migration as well as MMP-9 expression *in vitro*. In this experiment we succeeded in performing a xenograft mouse model using 4T1 cells within 8 days. We administered PGV-1, curcumin, or vehicle (corn oil) every two days for 12 days and evaluated the tumor progression (Figure 9). Tumor nodules were measurable, starting on day 8 after tumor xenograft. Tumor on vehicle-treated mice grew rapidly and reached tumor volume of ~600mm^3^ only in 4 days after tumor nodules appeared (day 12 after tumor injection). Curcumin slightly inhibits tumor progression, although not-significant statistically. On the other hand, PGV-1 significantly inhibited tumor progression compared to vehicle and curcumin treatments indicating that PGV-1 worked better in inhibiting tumor growth *in vivo* than curcumin.

## Discussion

This study was conducted to evaluate the tumor growth inhibitory activities of curcumin and its analogues, PGV-0, and PGV-1. At first, we conducted a new synthesis method for PGV-1 to improve the effectivity of the preparation by employing a high concentration of hydrochloric acid to accelerate the coupling reaction between electrophilic C-carbonyl at the benzaldehyde and the nucleophilic side at the cyclopentanone. This reaction seemed to work effectively to yield 50% PGV-1. Even though this result as not much as the previously reported by Sardjiman et al,^28^ but still comparable in the optimum yield and much more efficient with only 1h reaction. Therefore, this reaction method is recommended for further PGV-1 preparation.

For the anti-cancer evaluation, we used HER2-overexpressing breast cancer cells, MCF-7/HER2-5 for *in vitro* model, and then 4T1 cells for *in vivo* model. We found the IC_50_ value of curcumin analogues against MCF-7/HER2-5 cells was higher than that against MCF-7/mock cells, suggesting the HER2 expression alters in the cell sensitivity to curcumin and its analogues. Still, all the compounds exhibited cytotoxic activity with an IC_50_ value <20 µM, indicating the high potential of these compounds to be developed as chemotherapeutic agents. PGV-1 was found to be the most potent agent among the other compounds. However, in combination with doxorubicin, curcumin showed the maximum synergistic effect. The molecular mechanism of this combination needs to be elucidated further.

Since HER2 overexpression and NFκB overactivation are usually observed in cancer cells, both proteins have been considered as desirable targets for cancer therapy. Moreover, HER2 signaling is related to the NFκB pathway through PI3K/Akt signaling to activate IKK. Merhkofer et al^19^ reported that the IKK involved in HER2 signaling is IKKα, which functions to phosphorylate IκB, resulting in the degradation of IκB by the proteasome.^19^ Protein p65 that is released from IκB then translocated to the nucleus to act as a transcription factor. Protein p65 is a transcription factor for HER2 expression; hence, there is a loop-like HER2-NFκB-HER2 pathway that is a probable cause for breast cancer resistance to chemotherapeutic agents.^29^ In the present study, our data revealed that curcumin inhibited the localization of HER2. This result is not the first report showing that curcumin acts as a HER2 and a tyrosine kinase inhibitor. In 1999, a study conducted by Hong et al. also demonstrated the same result.^30^ However, the two curcumin analogues exhibited different activities on the localization of HER2. Based on their structure, the difference between PGV-0 and PGV-1 is due to the occurrence of methyl groups on the benzene ring (Figure 1), which may be responsible for their different mechanisms of action on cancer cells. This study revealed that PGV-0 acts like curcumin in inhibiting the localization of HER2, whereas PGV-1 did not have any effect on HER2. In addition, this *in vitro* assay confirmed our previous finding that PGV-1 exhibits the lowest interaction with EGFR and HER2 *in silico.*^9^ Another interesting finding in this study is that curcumin resulted in two additional bands with a higher molecular weight than the 185-kDa HER2. Given that these protein bands were found only in the curcumin-treated cells, it is possible that curcumin affects the post-translational modification of HER2. However, this hypothesis needs further investigation.

In this study, curcumin and PGV-0 were found to inhibit the translocation of p65 to the nucleus caused by doxorubicin, which might be related to their interaction with HER2. However, PGV-1 also inhibited the translocation of p65. There are other pathways in regulating of NFκB in addition to HER2 and PI3K/Akt, such as mitogen-activated protein kinase 1, NFκB-inducing kinase, NFκB-activating kinase, mixed-lineage kinase 3, and TGFβ-activated kinase 1.^31^ The effect of PGV-1 on other upstream molecules of NFκB is worth to be elucidated further. Another phenomenon found was that PGV-1 induced chromatin condensation in the nuclei (Figure 5G). This result is in agreement with our previous study showing that PGV-1 specifically induces prometaphase arrest during mitotic phase of cell cycle.^32^

Our study, together with other studies, demonstrated that curcumin and PGV-0 target the tyrosine kinase receptor and could block growth signaling in breast cancer, whereas PGV-1 acts directly in the nucleus to abrogate cell mitosis and decrease the localization of p65. These target mechanisms of PGV-1 may significantly contribute to its inhibitory effects of MMPs expression and cells migration which is important to be developed as anti-metastatic agent. In addition, PGV-1 was proven to work *in vivo* to significantly inhibit the tumor development of 4T1 implanted tumor model but not for curcumin. Since 4T1 cells is a TNBC model which is characterized as metastatic phenomena and supported with our previously finding,^12^ this *in vivo* data demonstrated that PGV-1 is suitable to treat more general metastatic cancers much better than curcumin. Overall, this study discovered that curcumin analogues exhibited a synergistic effect with doxorubicin on HER2-overexpressing breast cancer cells. Even that PGV-1 not specifically targets HER2, it still works better than curcumin to halt tumor development, including in *in vivo* study. Taken together with the strong cytotoxic effect of PGV-1 suggesting that this compound perhaps possesses some additional targets that differ to curcumin and PGV-0. In this regard, the cell cycle modulation may be the most interesting target of PGV-1 that should be more to pay attention.

## Conclusion

In conclusion, we showed curcumin and its analogues, PGV-0 and PGV-1 exhibit synergist cytotoxic effect with doxorubicin in HER2-overexpressed breast cancer cells. Curcumin and PGV-0 act at the upstream level of signaling cascade by inhibiting the localization of HER2 tyrosine kinase receptor, while PGV-1 acts at the downstream level by inhibiting nuclear translocation of NFκB subunit, p65. Moreover, PGV-1 show superior potency to inhibit cancer metastasis than curcumin. In *in vivo* experiment, PGV-1 exhibits more potent anticancer activity against TNBC tumor compared to curcumin; hence, highlighting the potency of PGV-1 to be considered as a chemopreventive agent for metastatic breast cancer.

## Ethical Issues

All animal experiments were performed in Animal Experimentation Facility of NAIST-Japan and the experimental procedures were approved by the Institutional Animal Care and Use Committee of NAIST (reference No. 1321) and the Ministry of Education, Culture, Sports, Science and Technology of Japan (Notice No. 71, 2006).

## Conflict of Interest

The authors declare that they have no competing financial interests.

## Acknowledgments

We express our gratitude to the International Collaboration Research Grant 2013-2014 Directorate General of Higher Education, Ministry of Education and Culture, Republic of Indonesia, No: 180/SP2H/PL/Dit.litabmas/IV/2012, who funded this research. We also thank Prof. Yoshio Inouye for the MCF7/HER2-5 cells used in this research.

## References

[1] Perrone D, Ardito F, Giannatempo G, Dioguardi M, Troiano G, Lo Russo L (2015). Biological and therapeutic activities, and anticancer properties of curcumin. Exp Ther Med.

[2] Deng YI, Verron E, Rohanizadeh R (2016). Molecular mechanisms of anti-metastatic activity of curcumin. Anticancer Res.

[3] Larasati YA, Yoneda-Kato N, Nakamae I, Yokoyama T, Meiyanto E, Kato JY (2018). Curcumin targets multiple enzymes involved in the ROS metabolic pathway to suppress tumor cell growth. Sci Rep.

[4] Nakamae I, Morimoto T, Shima H, Shionyu M, Fujiki H, Yoneda-Kato N (2019). Curcumin derivatives verify the essentiality of ROS upregulation in tumor suppression. Molecules.

[5] Prasad S, Tyagi AK, Aggarwal BB (2014). Recent developments in delivery, bioavailability, absorption and metabolism of curcumin: the golden pigment from golden spice. Cancer Res Treat.

[6] Da’i M, Yanuar A, Meiyanto E, Jenie UA, Supardjan AM (2013). (2E,5E)-2,5-Bis(4-hy-droxy-3-meth-oxy-benzyl-idene)cyclo-penta-none ethanol monosolvate. Acta Crystallogr Sect E Struct Rep Online.

[7] Sardjiman JJ. Synthesis of Some New Series of Curcumin Analogues, Antioxidative, Antiinflammatory, Antibacterial Activities and Qualitative-Structure Activity Relationships [thesis]. Yogyakarta: UGM Press; 2000.

[8] Meiyanto E, Supardjan DM, Agustina D (2006). Antiproliferative effect of pentagamavunon-0 on T47D breast cancer cells. Med J Yarsi.

[9] Meiyanto E, Putri DD, Susidarti RA, Murwanti R, Sardjiman Sardjiman, Fitriasari A (2014). Curcumin and its analogues (PGV-0 and PGV-1) enhance sensitivity of resistant MCF-7 cells to doxorubicin through inhibition of HER2 and NF-kB activation. Asian Pac J Cancer Prev.

[10] Ikawati M, Septisetyani EP (2018). Pentagamavunone-0 (PGV-0), a curcumin analog, enhances cytotoxicity of 5-fluorouracil and modulates cell cycle in WiDr colon cancer cells. Indones J Cancer Chemoprevent.

[11] Meiyanto E, Septisetyani EP, Larasati YA, Kawaichi M (2018). Curcumin analog pentagamavunon-1 (PGV-1) sensitizes Widr cells to 5-fluorouracil through inhibition of NF-κB activation. Asian Pac J Cancer Prev.

[12] Meiyanto E, Putri H, Arum Larasati Y, Yudi Utomo R, Istighfari Jenie R, Ikawati M (2019). Anti-proliferative and anti-metastatic potential of curcumin analogue, pentagamavunon-1 (PGV-1), toward highly metastatic breast cancer cells in correlation with ROS generation. Adv Pharm Bull.

[13] Iqbal N, Iqbal N (2014). Human epidermal growth factor receptor 2 (HER2) in cancers: overexpression and therapeutic implications. Mol Biol Int.

[14] Faltus T, Yuan J, Zimmer B, Krämer A, Loibl S, Kaufmann M (2004). Silencing of the HER2/neu gene by siRNA inhibits proliferation and induces apoptosis in HER2/neu-overexpressing breast cancer cells. Neoplasia.

[15] Sirkisoon SR, Carpenter RL, Rimkus T, Miller L, Metheny-Barlow L, Lo HW (2016). EGFR and HER2 signaling in breast cancer brain metastasis. Front Biosci (Elite Ed).

[16] Gupta P, Srivastava SK (2014). HER2 mediated de novo production of TGFβ leads to SNAIL driven epithelial-to-mesenchymal transition and metastasis of breast cancer. Mol Oncol.

[17] Harper KL, Sosa MS, Entenberg D, Hosseini H, Cheung JF, Nobre R (2016). Mechanism of early dissemination and metastasis in Her2(+) mammary cancer. Nature.

[18] Ithimakin S, Day KC, Malik F, Zen Q, Dawsey SJ, Bersano-Begey TF (2013). HER2 drives luminal breast cancer stem cells in the absence of HER2 amplification: implications for efficacy of adjuvant trastuzumab. Cancer Res.

[19] Merkhofer EC, Cogswell P, Baldwin AS (2010). Her2 activates NF-kappaB and induces invasion through the canonical pathway involving IKKalpha. Oncogene.

[20] Duru N, Candas D, Jiang G, Li JJ (2014). Breast cancer adaptive resistance: HER2 and cancer stem cell repopulation in a heterogeneous tumor society. J Cancer Res Clin Oncol.

[21] Bassères DS, Baldwin AS (2006). Nuclear factor-kappaB and inhibitor of kappaB kinase pathways in oncogenic initiation and progression. Oncogene.

[22] Bailey ST, Miron PL, Choi YJ, Kochupurakkal B, Maulik G, Rodig SJ (2014). NF-κB activation-induced anti-apoptosis renders HER2-positive cells drug resistant and accelerates tumor growth. Mol Cancer Res.

[23] Reynolds CP, Maurer BJ (2005). Evaluating response to antineoplastic drug combinations in tissue culture models. Methods Mol Med.

[24] Mahavorasirikul W, Viyanant V, Chaijaroenkul W, Itharat A, Na-Bangchang K (2010). Cytotoxic activity of Thai medicinal plants against human cholangiocarcinoma, laryngeal and hepatocarcinoma cells in vitro. BMC Complement Altern Med.

[25] Bravo-Cordero JJ, Hodgson L, Condeelis J (2012). Directed cell invasion and migration during metastasis. Curr Opin Cell Biol.

[26] Weaver AM (2006). Invadopodia: specialized cell structures for cancer invasion. Clin Exp Metastasis.

[27] Pulaski BA, Ostrand-Rosenberg S (2001). Mouse 4T1 breast tumor model. Curr Protoc Immunol.

[28] Sardjiman SS, Reksohadiprodjo MS, Hakim L, van der Goot H, Timmerman H (1997). 1,5-Diphenyl-1,4-pentadiene-3-ones and cyclic analogues as antioxidative agents Synthesis and structure-activity relationship. Eur J Med Chem.

[29] Cao N, Li S, Wang Z, Ahmed KM, Degnan ME, Fan M (2009). NF-kappaB-mediated HER2 overexpression in radiation-adaptive resistance. Radiat Res.

[30] Hong RL, Spohn WH, Hung MC (1999). Curcumin inhibits tyrosine kinase activity of p185neu and also depletes p185neu. Clin Cancer Res.

[31] Chen F, Demers LM, Shi X (2002). Upstream signal transduction of NF-kappaB activation. Curr Drug Targets Inflamm Allergy.

[32] Lestari B, Nakamae I, Yoneda-Kato N, Morimoto T, Kanaya S, Yokoyama T (2019). Pentagamavunon-1 (PGV-1) inhibits ROS metabolic enzymes and suppresses tumor cell growth by inducing M phase (prometaphase) arrest and cell senescence. Sci Rep.

